# Meta-analysis of randomized clinical trials comparing fistulectomy versus fistulotomy for low anal fistula

**DOI:** 10.1186/s40064-016-3406-8

**Published:** 2016-10-06

**Authors:** Yansong Xu, Siyuang Liang, Weizhong Tang

**Affiliations:** 1 Emergency Department, The First Affiliated Hospital of Guangxi Medical University, Nanning City, People’s Republic of China; 2 Colorectal and Anal Department, The First Affiliated Hospital of Guangxi Medical University, Nanning City, People’s Republic of China

**Keywords:** Fistulotomy, Fistulectomy, RCT, Anal fistula, Meta-analysis

## Abstract

**Objective:**

We evaluated the efficacy of fistulectomy compared to fistulotomy, and which procedure was the best procedure for patients with low anal fistula.

**Methods:**

The literature search included PubMed, EMBASE, Cochrane library, Google original studies and a manual search of reference on the topic of fistulectomy compared to fistulotomy for anal fistula that had a deadline for publication by June 2016. Randomized controlled trials studies were included in the review. The outcome variables were analyzed which including operative time, healing time, postoperative complications, recurrence and incontinence.

**Results:**

Six randomized controlled trials (fistulectomy = 280, fistulotomy = 285) were considered suitable for the meta-analysis, with a total of 565 patients. The result of meta-analysis indicated no statistically significant difference in operative time [OR 4.74, 95 % CI −2.74, 12.23, p = 0.21] and healing time [OR −3.32, 95 % CI −19.86, 13.23, p = 0.69] between the fistulectomy and fistulotomy procedures. Three main postoperative complications were included, and the combined result indicated no statistically significant difference in overall complications [OR 1.39, 95 % CI 0.51, 3.78, p = 0.52] and subgroup complication. At the end of follow up, two kinds of surgical methods have the same low recurrence rate and faecal incontinence. The result revealed that there was no significant difference in rate of fistula recurrence between the fistulectomy and the fistulotomy [OR 1.39, 95 % CI 0.70, 2.73, p = 0.34].

**Conclusion:**

The meta-analysis indicates that there is no conclusive evidence if fistulectomy or fistulotomy procedure is better in the treatment of low anal fistula.

## Background

Perianal fistulas remain a surgical treatment challenge in colorectal practice due to high recurrence rates and the risk of postoperative incontinence. This is indicated by several studies that report on incontinence, ranging from soiling to major incontinence, up to 41 % (van Koperen et al. 2008; Bokhari and Lindsey 2010). The most common treatment is represented by traditional fistulotomy because this is simple and gives good results, especially for low anal fistula. Fistulectomy is a valid alternative but, even if it’s more radical compared to traditional fistulotomy, is less used because of some disadvantages: longer operating time, wider surgical wound, prolonged time of healing and more than tripled incidence of incontinence to flatus (Wexner et al. 1996). Many present findings demonstrated fistulotomy resulted in lesser pain, bleeding, shorter wound healing time and shorter duration of postoperative wound discharge in comparison to a fistulectomy, but the current studies were mostly small samples and non-RCT researches (Parkash et al. 1985; Herold 2014; van der Hagen et al. 2006). Therefore, we designed this meta-analysis, and the purpose is to compare the advantages and disadvantages of two kinds of surgical procedures. Operating time, healing time and postoperative complications, recurrence and incontinence were collected and analyzed in this manuscript.

## Methods

### Search method

According to the preferred reporting items for systematic reviews and meta-analyses statement (PRISMA) guidelines (Moher et al. 2010). We searched the Medline, EMBASE, Cochrane Library and Google. The literature searches were carried out using medical subject headings and free-text word: anal fistula, perianal fistula, fistula in anal, fistulotomy and fistulectomy. Language is limited to English. Randomized controlled trials (RCT) comparing fistulotomy versus fistulectomy treatment in patients with low anal fistula were used to do a search strategy. Titles and abstract of studies identified by the search strategy were assessed in terms of their relevance and designed according to the selection criteria. Copies of all relevant and potentially relevant abstracts were obtained. If the studies met the inclusion criteria on initial assessment, full articles were obtained. This was repeated by another independent reviewer for verification. Any disagreement was resolved by further discussion.

### Inclusion criteria

All randomized controlled trials, which compared fistulotomy with fistulectomy treatment methods for low anal fistula, and which reported operative time, healing time, complications, recurrence and incontinence, were included.

### Exclusion criteria

Abstracts, letters, case reports, comments, and conference proceedings were excluded in the review. Studies on patients with complex anal fistula, intestinal tuberculosis, Crohn’s disease or infected with HIV who were treated by fistulotomy/fistulectomy and patients undergoing additional procedure along with fistulotomy/fistulectomy were also excluded from the study.

### Data collection

The primary reviewer (Yansong Xu) was responsible for extraction of details from eligible studies and summarizing the data using a data extraction sheet. The second reviewer (Weizhong Tang) then verified the extracted data. Two reviewers independently extracted the following from each study: operative time, healing time, complications, recurrence, incontinence.

### Statistical strategy

Dichotomous data are presented as the odds ratio (OR) and continuous outcomes as the weighted mean difference, both with 95 % CI. The overall effect was tested using Z scores and significance was set at p < 0.05. The meta-analysis was performed using fixed-effect or random-effect methods, depending on the absence or presence of significant heterogeneity. Statistical heterogeneity between trials was evaluated by the χ^2^ and I^2^ tests and significance was set at p < 0.10. In the absence of statistically significant heterogeneity, the fixed-effect method was used to combine the results. When heterogeneity was confirmed (p ≤ 0.10), the random-effect method was used. No sensitivity analysis was performed. Review manager 5.0 software was used. The quality of randomized clinical trials was the Jadad Scale (1996). Risk of bias summary was used to assess the risk of bias.

## Results

**Fig. 1:**
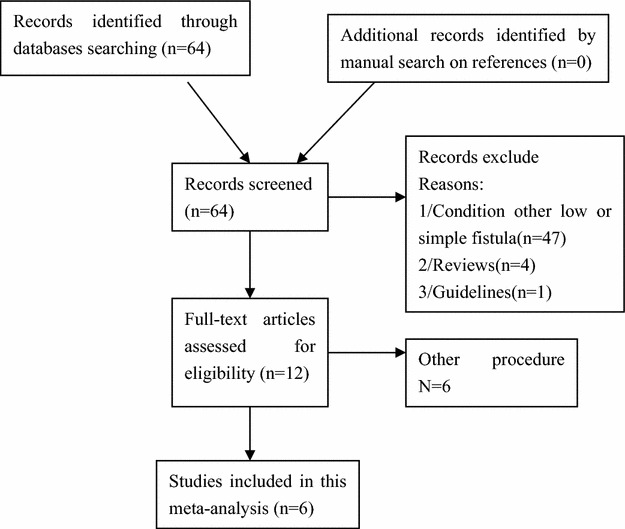
Flow diagram of the included literatures

Potentially relevant RCTs identified and screened for retrieval (n = 12), RCTs excluded because they compared fistulectomy to fistulectomy with marsupalization or other procedure (Jain et al. 2012; Chalya and Mabula 2013; Limongelli et al. 2016; Pescatori et al. 2006; Ho et al. 1998; Toyonaga et al. 2007). Finally, 6 RCT publications which involved 565 patients fulfilled the inclusion criteria and were included in this review (Gafar 2013; Nazzer et al. 2012; Bhatti and Fatima 2011; Sheikh and Shukr 2015; Filingeri et al. 2004; Kronborg 1985) (Fig. 1). Table 1 shows the basic characteristic of included studies. Table 2 shows postoperative results.

**Table 1:** The basic characteristic of included studies

First author	Year	Country	GroupA/group B	Follow-up period (month)	Quality of the study
Gafar (2013)	2013	Egypt	18/18	4	5
Nazeer (2012)	2012	Pakistan	75/75	10	4
Bhatti (2011)	2011	Pakistan	25/25	6	5
Sheikh (2015)	2015	Pakistan	131/131	6	7
Filigeri (2004)	2004	Italy	10/10	6	6
Kronborg (1985)	1985	Denmark	20/17	12	5

*I* infection, *B* bleeding, *P* pain, *NA* not available, *Group A* fistulectomy, *Group B* fistulotomy

**Table 2:** The postoperative characteristic of included studies

Author	Operative time (mean), minutes		Healing time (mean), days		Recurrence		Complication		Incontinence	
	Group A	Group B	Group A	Group B	Group A	Group B	Group A	Group B	Group A	Group B
Gafar (2013)	15.9 ± 0.9	13.9 ± 0.7	26.6 ± 1.4	21.0 ± 3.0	1	0	0I	1I	NA	NA
Nazeer (2012)	NA	NA	NA	NA	0	0	15P, 5B	9P, 1B	0	0
Bhatti (2011)	NA	NA	NA	NA	0	0	7P, 3B	3P, 1B	0	0
Sheikh (2015)	25.9 ± 3.6	14.2 ± 3.2	32.0 ± 3.5	28.3 ± 2.3	20	14	3I, 1B, 97P	5I, 4B, 101P	NA	NA
Filigeri (2004)	18.3 ± 2.9	17.9 ± 2.8	24.5 ± 4.9	41.3 ± 7.3	NA	NA	0B	0B	0	0
Kronborg (1985)	NA	NA	NA	NA	2	3	NA	NA	3	1

*Group A* fistulectomy; *Group B* fistulotomy

### Operative time

Operative time was only reported in three studies (Gafar 2013; Sheikh and Shukr 2015; Filingeri et al. 2004). As statistically significant heterogeneity was evident for the outcome measure of operative time, the random effect model was used to combine the data. The findings indicated that no significant difference was showed, when primary fistulectomy or fistulotomy procedure was performed for low anal fistula [OR 4.74, 95 % CI −2.74, 12.23, p = 0.21].

### Healing time

Healing time was only reported in three studies (Gafar 2013; Sheikh and Shukr 2015; Filingeri et al. 2004). Significant heterogeneity was detected (p < 0.00001, I^2^ = 96 %), using the random-effect method for the meta-analysis. The result of meta-analysis indicated no statistically significant difference in healing time between the fistulectomy and fistulotomy procedure [OR −3.32, 95 % CI −19.86, 13.23, p = 0.69].

### Complications

Five articles reported on different complications (Gafar 2013; Bhatti and Fatima 2011; Sheikh and Shukr 2015; Filingeri et al. 2004). The main complications included wound pain, wound infection and wound bleeding. Hence, subgroup analysis was done for the outcome measure of each complication. The fixed effect model was used for different complication, respectively, which did not show an advantage for either technique concerning postoperative wound pain[OR 1.17 (95 % CI 0.75, 1.83), p = 0.49], postoperative wound infection[OR 0.53 (95 % CI 0.14, 1.97), p = 0.34], postoperative wound bleeding [OR 1.52 (95 % CI 0.53, 4.32), p = 0.43] and overall complication [OR 1.39 (95 % CI 0.51, 3.78), p = 0.52].

### Recurrence rate

Five articles reported on recurrence at the end of follow-up ranged 4 to 12 months (Gafar 2013; Nazzer et al. 2012; Bhatti and Fatima 2011; Sheikh and Shukr 2015; Kronborg 1985). According to the results of the heterogeneity analysis (p = 0.55, I^2^ = 0 %), using the fixed-effect method for the meta-analysis. The combined result indicated no statistically significant difference in the recurrence rate between the fistulectomy and fistulotomy procedure [OR 1.39, 95 % CI 0.70, 2.73, p = 0.34].

### Postoperative faecal incontinence

Four articles reported on postoperative incontinence in this meta-analysis (Nazzer et al. 2012; Bhatti and Fatima 2011; Filingeri et al. 2004; Kronborg 1985). Three patients (of the 21 patients) who underwent primary fistulectomy procedure in the study by Bhatti et al. developed incontinence to liquid stools, compared with one patient (of the 26 patients) in the group undergoing fistulotomy. None of the other studies reported any cases of incontinence to liquid or solid stools.

### Assess the risk of bias

**Fig. 2:**
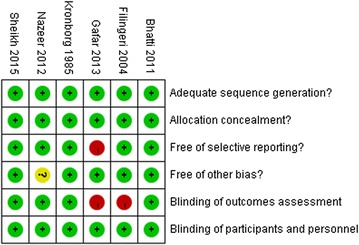
Risk of bias summary

The judgements about each risk of bias item for each included study was showed low risk of bias (Fig. 2).

## Discussion

It is controversial whether to perform a fistulotomy or a fistulectomy for a low anal fistula. In fistulectomy, the whole track and adjacent tissue is removed resulting in larger wound followed by more risk of postoperative pain, bleeding and wound infection with longer healing time. In fistulotomy, lesser amount of tissue is excised resulting in earlier healing time as compared to fistulectomy (Isbister 1999). This paper only has compared the clinical effect of two surgical procedures in low anal fistula. We compared the operating time, healing time and complications, recurrences, faecal incontinence.

The first randomized controlled trial comparing fistulotomy to fistulectomy in a small sample by Kronborg 1985 demonstrated that healing time was significantly quicker for fistulotomy (p < 0.01), while recurrence was similar (2/21 vs. 3/24) at 1 year (Kronborg 1985), and there was no statistically significant difference in the recurrence rate. The results of Kronborg were consistent with Sheikh et al., but Sheikh et al. considered the fistulotomy could short the operative time and speed up the patient’s recovery (Sheikh and Shukr 2015). This observation can be explained by the fact that the fistulectomy operation requires dissection of the fistula tract from the surrounding tissues, followed by coagulation of bleeding to control homeostasis. During a fistulotomy, the fistula tract is laid open, so dissection of the fistula tract is not required. Thus fistulectomy procedure is likely to take a longer operative time. In addition, Filigeri described that the application of radiofrequencies to fistulectomy renders more feasible, because radiofrequency fistulectomy significantly shortened the operative time, but there was no a statistically significant difference in the operative time (Filingeri et al. 2004).

The American Society of Colon and Rectal Surgeons guidelines (Whiteford et al. 2005) quote recurrence and incontinence rates from old retrospective studies with 0–9 % recurrence after fistulotomy, and incontinence risk 0–28 % (Göttgens et al. 2015; Whiteford et al. 2005). ASCRS states that fistulectomy has a triple incidence of transient flatus incontinence compared to fistulotomy (Wexner et al. 1996). In fact, we observed that the incidence of this complication is almost the same in the two groups. This combined result indicated no statistically significant difference in the recurrence rate between the fistulectomy and fistulotomy procedures. There are many factors that affect the postoperative results.

Our study demonstrated that fistulotomy was associated with significantly lesser postoperative pain, infection and bleeding as compared to the fistulectomy, but these differences did not reach statistical significance. As is well known, clinicians are using electric knife skillfully and wildly which can ensure less bleeding during operation. Most of the patients were treated with cleaning enema before surgery. Hence, the postoperative complications of the two operative procedures were relatively low. Only a clinical trial has demonstrated the development of anal incontinence after fistulectomy and fistulotomy in the treatment of low fistula-in-ano during 12 month follow-up period (Gafar 2013; Bhatti and Fatima 2011; Filingeri et al. 2004; Kronborg 1985) which the follow-up time was longer than that of others. For patients, the long-term outcome was concerned. So, Reasonable follow-up time can increase the reliability of the conclusion. A mean 7.8 years follow-up after surgery for simple and complex cryptoglandular fistulas pointed out that surgical fistulotomy was the strongest risk factor for fecal incontinence (Visscher et al. 2015). The follow-up time ranged 4–12 months in this meta-analysis. The follow-up time was too short to be not conducive to the authenticity and accuracy of the results, so longer follow-up should be considered. The severity of incontinence increaseed with the complexity of the fistula, negatively influencing quality of life (Novotny et al. 2008). Göttgens reported a minimal effect on continence status after fistulotomy procedure after 5 years (Göttgens et al. 2015). Gafar et al. (2013). Reported that fistulotomy is simple and results in shorter operative time, lesser recurrence rate, and earlier wound healing as compared with fistulectomy. Children’s anal fistula is rare, and treatment of fistula in ano in infant remains controversial, but we believe that children should take a small injury, a short time of operation. Novotny et al. (Ratto et al. 2015). reported that recurrence is more likely in older children and in children who had previous episodes of PAA or if pus was noted at the time of surgery. In Filingeri et al. (2004) study, we found that authors operated only submucosal fistulas, according to Goodsall’s rule, that does not involve the sphincter. So, we did not find complication or recurrence, incontinence. A recent systematic review showed that fistulotomy or fistulectomy and primary sphincteroplasty could be a therapeutic option for complex anal fistula after a long follow-up, with a success rate of about 90 %, and the success rate was not related to either the type of fistula excision or sphincter reconstruction modality (Ratto et al. 2015).

This is the first meta-analysis which comparing fistulectomy versus fistulotomy for low anal fistula. Selection bias may influence the results and conclusion of the meta-analysis. Firstly, the main limitation of our study is the small number of randomized controlled studies. Secondly, perhaps, there are many more unpublished articles that often cannot be accessed. Thirdly, in order to better reflect the objectivity, and to adapt to more readers to read, we limited English language. In spite of the limitations of the study, meta-analysis can provide increased statistical power to detect small effect sizes, and is more able to show the accuracy and reliability of the study.

In brief, according to the meta-analysis, there is no conclusive evidence to show which method is better for simple anal fistula. Future randomized trials when pooled further in the meta-analysis may answer this question.

## References

[CR1] Bhatti Y, Fatima S (2011). Fistulotomy versus fistulectomy in the treatment of low fistula in ano. Rawal Med J.

[CR2] Bokhari S, Lindsey I (2010). Incontinence following sphincter division for treatment of anal fistula. Colorectal Dis.

[CR3] Chalya PL, Mabula JB (2013). Fistulectomy versus fistulotomy with marsupialisation in the treatment of low fistula-in- ano: a prospective randomized controlled trial. Tanzan J Health Res.

[CR4] Filingeri V, Gravante G, Baldessari E, Casciani CU (2004). Radiofrequency fistulectomy vs. diathermic fistulotomy for submucosal fistulas: a randomized trial. Eur Rev Med Pharmacol Sci.

[CR5] Gafar AA (2013). Fistulotomy versus fistulectomy as a treatment for low anal fistula in infants: a comparative study. Ann Pediatr Surg.

[CR6] Göttgens KWA, Janssen PTJ, Heemskerk J, van Dielen FMH, Konsten JLM, Lettinga T (2015). Long-term outcome of low perianal fistulas treated by fistulotomy: a multicenter study. Int J Colorectal Dis.

[CR7] Herold A, Abcarian H (2014). Fistulectomy with primary sphincter reconstruction. Principles and management.

[CR8] Ho YH, Tan M, Leong AFPK, Seow-Choen F (1998). Marsupialization of fistulotomy wounds improves healing: a randomized controlled trial. Br J Surg.

[CR9] Isbister WH (1999). Fistula-in-Ano. Aust N Z J Surg.

[CR10] Jadad AR, Moore RA, Carroll D, Jenkinson C, Reynolds DJM, Gavaghan DJ (1996). Assessing the quality of reports of randomized clinical trials: is blinding necessary?. Control Clin Trials.

[CR11] Jain BK, Vaibhaw K, Garg PK, Gupta S, Mohanty D (2012). Comparison of a fistulectomy and a fistulotomy with marsupialization in the management of a simple anal fistula: a randomized, controlled pilot trial. J Korean Soc Coloproctol.

[CR12] Kronborg O (1985). To lay open or excise a fistula-in-ano: a randomized trial. Br J Surg.

[CR13] Limongelli P, Brusciano L, del Genio G, Tolone S, Bosco A, Docimo G (2016). Marsupialization compared to open wound improves dressing change and wound care management after fistulectomy for low transsphincteric anal fistula. Int J Colorectal Dis.

[CR14] Moher D, Liberati A, Tetzlaff J, Altman DG, PRISMA Group (2010). Preferred reporting items for systematic reviews and meta-analyses: the PRISMA statement. Int J Surg.

[CR15] Nazzer MA, Saleem R, Ali M (2012). Better option for the patients of low fistula in ano: fistulectomy or fistulotomy. Pain.

[CR16] Novotny NM, Mann MJS, Rescorla FJ (2008). Fistula in ano in infants: who recurs?. Pediatr Surg Int.

[CR17] Parkash S, Lakshmiratan V, Gajendran V (1985). Fistula-in-ano: treatment by fistulectomy, primary closure and reconstitution. Aust N Z J Surg.

[CR18] Pescatori M, Ayabaca SM, Cafaro D, Iannello A, Magrini S (2006). Marsupialization of fistulotomy and fistulectomy wounds improves healing and decreases bleeding: a randomized controlled trial. Colorectal Dis.

[CR19] Ratto C, Litta F, Donisi L, Parello A (2015). Fistulotomy or fistulectomy and primary sphincteroplasty for anal fistula (FIPS): a systematic review. Tech Coloproctol.

[CR20] Sheikh IA, Shukr I (2015). Fistulotomy vs fistulectomy in the treatment of simple low anal fistula of male patients. Pak Armed Forces Med J.

[CR21] Toyonaga T, Matsushima M, Tanaka Y, Suzuki K, Sogawa N, Kanyama H (2007). Non-sphincter splitting fistulectomy vs conventional fistulotomy for high trans-sphincteric fistula-in-ano: a prospective functional and manometric study. Int J Colorectal Dis.

[CR22] Van der Hagen SJ, Baeten CG, Soeters PB, Soeters PB, Van Gemert WG (2006). Long-term outcome following mucosal advancement flap for high perianal fistulas and fistulotomy for low perianal fistulas: recurrent perianal fistulas: failure of treatment or recurrent patient disease?. Int J Colorectal Dis.

[CR23] van Koperen PJ, Wind J, Bemelman WA, Bakx R, Reitsma JB, Slors JF (2008). Long-term functional outcome and risk factors for recurrence after surgical treatment for low and high perianal fistulas of cryptoglandular origin. Dis Colon Rectum.

[CR24] Visscher AP, Schuur D, Roos R, van der Mijnsbrugge GJ, Meijerink WJ, Felt-Bersma RJ (2015). Long-term follow-up after surgery for simple and complex crypto glandular fistulas: fecal incontinence and impact on quality of life. Dis Colon Rectum.

[CR25] Wexner SD, The Standards Practice Task Force, The American Society of Colon and Rectal Surgeons (1996). Practice parameters for treatment of fistula-in-ano–supporting documentation. Dis Colon Rectum.

[CR26] Whiteford MH, Kilkenny J, Hyman N, Buie WD, Cohen J, Orsay C (2005). Practice parameters for the treatment of perianal abscess and fistula-in-ano (revised). Dis Colon Rectum.

